# Vaccine Hesitancy on Social Media: Sentiment Analysis from June 2011 to April 2019

**DOI:** 10.3390/vaccines9010028

**Published:** 2021-01-07

**Authors:** Hilary Piedrahita-Valdés, Diego Piedrahita-Castillo, Javier Bermejo-Higuera, Patricia Guillem-Saiz, Juan Ramón Bermejo-Higuera, Javier Guillem-Saiz, Juan Antonio Sicilia-Montalvo, Francisco Machío-Regidor

**Affiliations:** 1 Department of Preventive Medicine and Public Health, Bromatology, Toxicology and Legal Medicine, University of Valencia, 46010 Valencia, Spain; 2 Faculty of Engineering and Technology, International University of La Rioja, 26006 Logroño, Spain; diego.piedrahita@comunidadunir.net (D.P.-C.); javier.bermejo@unir.net (J.B.-H.); juanramon.bermejo@unir.net (J.R.B.-H.); juanantonio.sicilia@unir.net (J.A.S.-M.); francisco.machio@unir.net (F.M.-R.); 3 Department of Preventive Dentistry, Epidemiology and Public Health, European University of Valencia, 46010 Valencia, Spain; patricia.guillem@universidadeuropea.es; 4 CIBER in Physiopathology of Obesity and Nutrition (CIBERobn), Institute of Health Carlos III, 28029 Madrid, Spain; 5 Department of Psychology, International University of Valencia, 46002 Valencia, Spain; javier.guillem@campusviu.es

**Keywords:** vaccine hesitancy, vaccination, opinion mining, sentiment analysis, content analysis, machine learning, social media, Twitter

## Abstract

Vaccine hesitancy was one of the ten major threats to global health in 2019, according to the World Health Organisation. Nowadays, social media has an important role in the spread of information, misinformation, and disinformation about vaccines. Monitoring vaccine-related conversations on social media could help us to identify the factors that contribute to vaccine confidence in each historical period and geographical area. We used a hybrid approach to perform an opinion-mining analysis on 1,499,227 vaccine-related tweets published on Twitter from 1st June 2011 to 30th April 2019. Our algorithm classified 69.36% of the tweets as neutral, 21.78% as positive, and 8.86% as negative. The percentage of neutral tweets showed a decreasing tendency, while the proportion of positive and negative tweets increased over time. Peaks in positive tweets were observed every April. The proportion of positive tweets was significantly higher in the middle of the week and decreased during weekends. Negative tweets followed the opposite pattern. Among users with ≥2 tweets, 91.83% had a homogeneous polarised discourse. Positive tweets were more prevalent in Switzerland (71.43%). Negative tweets were most common in the Netherlands (15.53%), Canada (11.32%), Japan (10.74%), and the United States (10.49%). Opinion mining is potentially useful to monitor online vaccine-related concerns and adapt vaccine promotion strategies accordingly.

## 1. Introduction

The Strategic Advisory Group of Experts (SAGE) Working Group on Vaccine Hesitancy defines vaccine hesitancy as the “delay in acceptance or refusal of vaccination despite the availability of vaccination services. Vaccine hesitancy is complex and context-specific, varying across time, place, and vaccines. It is influenced by factors such as complacency, convenience, and confidence” [1]. The World Health Organisation included vaccine hesitancy in the list of the ten major threats to global health in 2019 [2].

Digital networks could contribute to the increase in vaccine hesitancy since they allow the fast spread of rumours and myths regarding vaccination [3]. Moreover, the information available on the World Wide Web could influence people’s decision to accept, delay or refuse vaccination [4,5], and this may have facilitated outbreaks of vaccine-preventable diseases among unvaccinated populations [3]. The emergence of the Internet as a key source of vaccine-related content [6,7] has made it essential to incorporate online information monitoring in the strategies for addressing vaccine hesitancy. In the last decade, social networks have become an important tool for opinion research. Unlike other online sites, social media sites allow users from different countries to have public discussions about any topic, including vaccination, in real-time. Besides, public health professionals have also taken an active role in these conversations. Consequently, social media is not only a platform for real-time surveillance of vaccine-hesitancy and infectious diseases but also a useful communication tool for global health actors [8].

The vaccine hesitancy monitorisation could include text mining to extract data from social media messages, analyse it, classify the stance towards vaccination [9] and detect the main subtopics of concern. Sentiment analysis (SA) [10] is the text-mining subfield that allows the classification of opinions according to the polarity (positive, negative, or neutral) [11], the emotion (happiness, sadness, fear, etc.) [12], or the intensity of agreement based on a numerical rating scale [13]. SA can be performed at three levels [14]: (a) document-level SA, performed to find out the global opinion of a document; (b) sentence-level SA, which uncovers the attitude expressed by each sentence; (c) aspect-level SA, which classifies the opinion towards an entity.

There are three types of SA approaches [14]: lexicon-based approaches, machine-learning approaches, and hybrid approaches—a combination of lexicon and machine-learning approaches. Lexicon-based approaches rely on corpora or dictionaries that contain terms classified by their sentiment [15]. Their algorithms assign a sentiment score to each unit of the text using the lexicon as a reference. These scores are aggregated to calculate the predominant sentiment in the text [16,17]. Within machine-learning approaches, there are three methods. Supervised learning techniques train their classification algorithms with data that were labelled previously by the investigators. They are useful for predicting outcomes about a text with pre-established categories [18]. Among the supervised machine learning techniques, the support vector machine (SVM) approach has the highest accuracy [19]. On the other hand, unsupervised machine learning algorithms find new patterns [18] in unlabelled data [20]. Lastly, semi-supervised learning methods use labelled and unlabelled data to design the most appropriate classification model [21].

Most of the qualitative and quantitative health research in social networks is focused on Twitter [22,23] due to the ease of obtaining a large volume of public data [22] and the length limitation of each message (tweet) [23]. Basic information about Twitter and the definitions of the Twitter terms mentioned in this article can be found in Appendix A. Published articles about opinion analysis towards vaccination on social media usually perform short-term SA [9,24,25] in small datasets [24,26]. Some studies are limited to a single location [24,25,27], but usually geolocation is not analysed [3]. In our research, we aim to evaluate public perceptions regarding vaccination on Twitter by performing a sentence-level SA on a dataset composed of 1,499,227 vaccine-related tweets, in English and Spanish, published from June 2011 to April 2019. We used a hybrid approach, as its performance is superior to independently applied lexicon-based approaches and machine-learning approaches [28].

## 2. Materials and Methods

### 2.1. Data Source and Data Extraction

Public Twitter messages containing at least one vaccination keyword (Appendix B) were collected prospectively by using the Twitter Application Programming Interface (API) between 18 March 2019 and 15 April 2019. From this set, we selected twenty-eight vaccine-related hashtags (Appendix C). Afterwards, we conducted a retrospective search of all public tweets (excluding retweets) posted from 1 June 2011 to 30 April 2019 that mentioned those hashtags, without language limitations. The search returned 1,652,156 tweets from 307,682 users. For each tweet, we stored the text and some related metadata (date of publication, retweet status, number of retweets, number of favourites or likes, number of replies, language, tweet ID, user ID, user geolocation if available, and user-reported profile location).

### 2.2. Data Preparation

After data collection, we removed tweets written in languages other than English or Spanish and tweets that only contained URLs or hashtags. The final database contained 1,499,227 original tweets from 258,904 users.

Next, tweet ID and user ID were anonymised with data masking and the usernames mentioned in the tweets were replaced with the code ‘user_mention’. To establish the location of each tweet at the country level, we used the user geolocation and the user-reported profile location. In addition, we created a hashtag database.

### 2.3. Sentiment Polarity Analysis

The polarity analysis was performed using a hybrid model based on the combination of a lexicon-based approach and a supervised machine-learning approach. Two independent coders manually annotated a simple random sample of original tweets for sentiment polarity (positive, negative, or neutral) (Appendix D). The intercoder reliability was high (Cohen’s Kappa score > 0.82 for each category). The two researchers discussed their results to resolve discrepancies.

Before we trained our model, text pre-processing was performed in all the dataset by taking the following steps:

Remove punctuation symbols, URLs, emoticons, hash (#) symbol, and special characters.

Convert all characters to lowercase.

Replace two or more spaces with a single space.

Tokenisation: Divide the phrases into words.

Spelling correction: Remove additional letters.

Stop words removal: Eliminate articles, prepositions, conjunctions, etc.

Lemmatisation: Transform the inflected forms of the words into their lemma, the dictionary form, in order to group the words with the same meaning.

After pre-processing the tweets, 5000 tweets of each class were randomly selected to build the classifier. Then, these data were split into a training set (3500 tweets of each class) and a testing set (2500 tweets of each class). We performed a syntactic and semantic analysis of the training data. Then, we created a domain-specific sentiment polarity dictionary. We also assigned a polarity to the most frequent hashtags in our hashtag database. All this information was used as a feature input to train the SVM classifier. The performance of the algorithm was evaluated on the testing set. The identified errors were corrected, and the process was iteratively conducted until the algorithm achieved an accuracy rate > 85% for the three classes.

### 2.4. Statistical Analysis

The descriptive analysis (absolute and relative frequencies, mean and median as measures of central tendency, standard deviation and range as measures of dispersion) was performed on IBM Statistical Package for the Social Sciences (SPSS) for Windows, version 23.0 (IBM Corp., Armonk, NY, USA). The historical trend in each series was evaluated by fitting linear, exponential, quadratic, cubic, and logistic models to the data and selecting the best fitting curve. One-way ANOVA, Kruskal–Wallis, and post-hoc tests were conducted to compare means and medians among three or more categories. The significance level was set at 0.05.

### 2.5. Ethical Statement

This research received ethical approval by the Committee of Ethics and Human Research of the University of Valencia. Even though we used public data available on Twitter and not human subject data, the information was anonymised, and the messages were removed from the final database to protect the identity of the authors. Informed consent was not feasible because of the nature of the study.

## 3. Results

We analysed 1,295,823 (86.43%) tweets in English and 203,404 (13.57%) tweets in Spanish. A total of 89,186 (5.95%) institutional tweets were identified. There were 2700 vaccine-related tweets in June 2011. The number of tweets showed an increasing monthly trend (*p* = 0.00, R^2^ = 0.7752, exponential model) until April 2015, with a peak of 57,544 tweets in February 2015. This was followed by a decreasing trend until December 2015 (*p* = 0.00, R^2^ = 0.8083, exponential model). Then, the monthly number of tweets oscillated between 13,569 and 22,296, with rising points every April. Lastly, we observed a mean increase of 8952 tweets per month (95% CI 2632–15,272 tweets per month) from December 2018 to April 2019 (increasing trend, *p* = 0.00, R^2^ = 0.9929, exponential model), reaching the highest peak (57,667 tweets) in the last month of this study.

**Figure 1:**
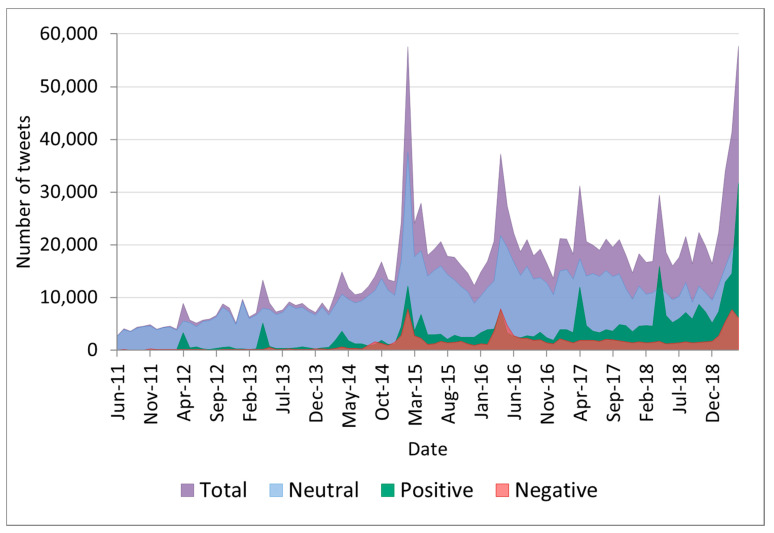
Area chart for the number of tweets classified by sentiment (June 2011–April 2019).

The algorithm classified 1,039,864 (69.36%) tweets as neutral, 326,497 (21.78%) tweets as positive and 132,866 (8.86%) tweets as negative. The distribution of tweets per sentiment throughout the study period is shown in Figure 1.

**Figure 2:**
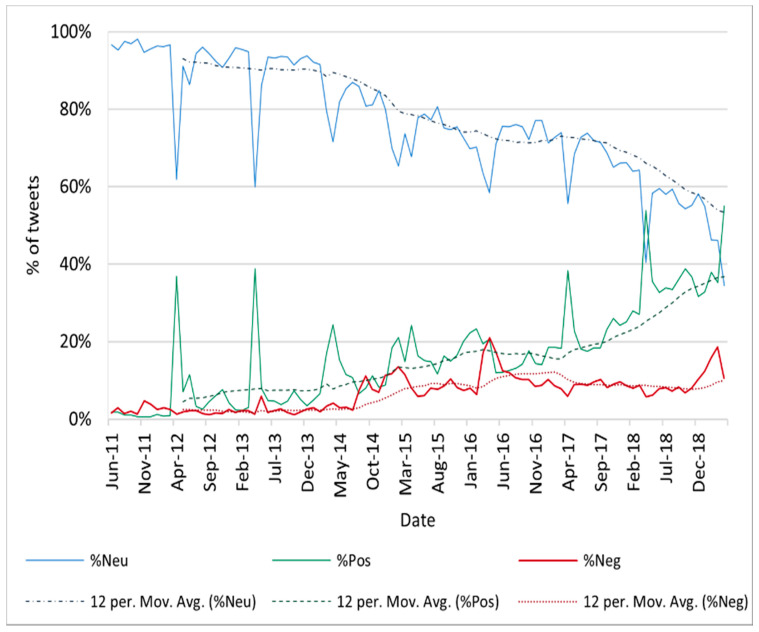
Relative frequency of tweets classified as neutral, positive, and negative (June 2011–April 2019).

The relative frequency of neutral tweets showed a decreasing tendency in the study period (*p* = 0.00, R^2^_neutral group_ = 0.7479, linear model), while the proportion of positive tweets and the proportion of negative tweets increased (*p* = 0.00, R^2^_positive group_ = 0.6902 and R^2^_negative group_ = 0.6187, exponential model) (Figure 2). In April 2018 and April 2019, the proportion of neutral tweets dropped to a minimum of 40.47% and 34.51%, respectively, while the proportion of positive tweets exceeded 50% (53.81% and 54.97%, respectively. The negative group only accounted for a percentage of more than 20% of the dataset once, in April 2016 (20.96%).

An analysis of the monthly absolute and relative number of tweets per sentiment polarity was performed. The mean of total tweets and positive tweets was higher in April (27,545 and 10,736 tweets, respectively). The difference between April and the other months was statistically significant (*p* < 0.05) in every case, except for the global average in February (*p* = 0.209) and March (*p* = 0.092), and for the average of positive tweets in February (*p* = 0.070). The monthly mean of negative and neutral tweets did not show any statistically significant difference. Nonetheless, data were highly dispersed and the monthly median of total tweets and tweets by sentiment polarity showed no monthly statistically significant differences. The percentage of neutral tweets per month was significantly lower in April (56.27%), while the percentage of positive tweets was significantly higher (36.47%). There were no statistically significant differences in the monthly percentage of negative tweets.

The median of total tweets and tweets by sentiment polarity was significantly higher (*p* < 0.05) every weekday than on Saturdays and Sundays. The average proportion of negative tweets was lower on Wednesdays (7.95%), and higher on Saturdays (10.76%) and Sundays (11.93%). The average proportion of positive tweets followed the opposite pattern. It was higher on Wednesdays (23.25%), and lower on Saturdays (20.42%) and Sundays (19.41%). The proportion of neutral tweets oscillated between 68.66% and 70.14%, with no significant variation between weekdays and weekends (*p* = 0.070).

The number of posts per user ranged from 1 to 21,329 tweets (median of 1 tweet/user, mean of 5, 24 tweets/user). A total of 68,801 (26.57%) users tweeted two or more vaccine-related messages containing the selected hashtags. In this group, 63,181 (91.83%) users had a homogeneous sentiment polarity towards vaccination in all their tweets: 52,020 (75.61%) users were totally neutral, 9626 (13.99%) totally positive and 1535 (2.23%) totally negative. On the other hand, 5620 (8.17%) users expressed different attitudes. Specifically, 4764 (6.92%) users had both positive and negative tweets towards vaccination, and they authored 472,959 tweets (31.55% of the dataset).

The retweet and favourites/likes frequencies were statistically significantly different among the three sentiment polarity categories. Positive tweets had a higher mean of retweets (4.99) than negative (2.17) and neutral tweets (1.37). Positive tweets also had a larger number of favourites/likes (7.97) than negative (2.34) and neutral tweets (1.52). As for the number of replies, it showed no statistically significant differences between the positive (0.42) and the negative tweets (0.39). Both groups received more replies than neutral tweets (0.19).

**Figure 3:**
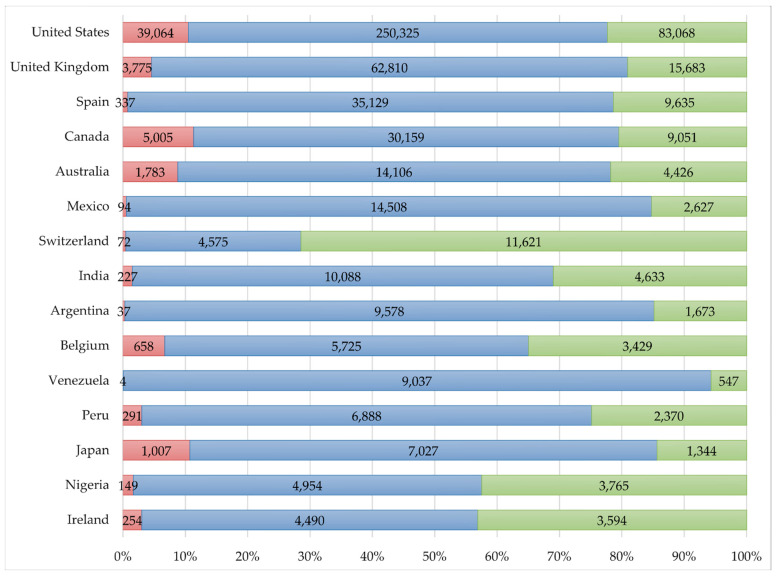
Absolute and relative number of tweets by sentiment polarity and location (June 2011–April 2019).

Geolocation was available for 144,651 (55.87%) users, responsible for 779,430 (51.99%) tweets. We retrieved tweets from every country except Georgia, Libya, Suriname, and Zimbabwe. A total of 41 countries had more than 1000 tweets each, and a total of 751,755 (50.14%) tweets were written in these locations. The greatest proportion of negative posts was registered in the Netherlands (n _negative_ = 897, 15.53%), Canada (n _negative_ = 5005, 11.32%), Japan (n _negative_ = 1007, 10.74%) and the United States (n _negative_ = 39,064, 10.49%). Two countries had a proportion of positive tweets higher than 50%: Switzerland (n _positive_ = 11,621, 71.43%) and Kenya (n _positive_ = 2030, 53.55%). The rate of positive tweets was also significantly higher in Nigeria (n _positive_ = 3765, 42.46%), Ireland (n _positive_ = 3594, 43.10%), Puerto Rico (n _positive_ = 850, 39.89%), Pakistan (n _positive_ = 671, 36.87%), Belgium (n _positive_ = 3429, 34.95%), and Turkey (n _positive_ = 824, 34.28%). The absolute and relative frequencies of each sentiment in the 16 locations with more tweets are represented in Figure 3.

## 4. Discussion

To our knowledge, this is one of the few studies to analyse sentiment polarity towards vaccination in more than one million tweets written over eight years and the only one to do it at sentence level using a hybrid approach.

We observed a growing interest in vaccines over time, partially explained by the parallel growth in the number of Twitter users from 2011 to 2019. The highest number of vaccine-related tweets was detected in April 2019, as we included the hashtags used for the annual Immunization Weeks. The second highest peak was identified in February 2015, concurrent with a mediatic political debate over mandatory vaccination in the United States. Despite most of the tweets in our database being neutral, the percentage of negative and positive tweets showed an increasing tendency throughout the entire study period. Negative tweets never became predominant over neutral tweets, but they were more frequent than positive tweets on three periods: (1) Between July 2011 and March 2013 (+1.5% average), (2) December 2014 (+2.49%), (3) April 2016–June 2016 (+5.01% in May). The proportion of negative tweets remained under 20% along all the study period, except in April 2016, simultaneously with a controversy raised by a documentary that linked vaccines and autism. Positive tweets were predominant in the dataset twice, in April 2018 and April 2019 (+13.34% and +20.46% higher than neutral tweets, respectively).

We detected relative and absolute peaks in positive tweets in April, the month in which World Immunisation Week and the European Immunisation Week are celebrated. However, we did not find any impact on the frequency of negative tweets. This could be explained by the echo-chamber effect, in which people are more likely to interact with users that share their opinion towards vaccines [29]. They tend to ignore information that contains opposite attitudes and beliefs [30]. In consequence, most users only consume homogeneously polarised vaccine-related content. They separate into polarised groups that barely communicate with each other and, when they do, they engage in arguments that reinforce their previous ideas about vaccination [30]. For this reason, some authors [29,31,32] suggest that current public health interventions may not improve vaccination acceptance and, in some cases, they could reinforce vaccinate hesitancy. Instead, other strategies should be explored, such as using hashtags and keywords that hesitant users usually search [33], posting messages with moderate risk negations, rather than extreme risk negations [34], encourage the participation of community members as spokespeople for the immunisation campaigns, and engage in direct conversations with hesitant people aiming to understand the motives of their doubts and build their trust.

As for the relative frequency of tweets by day of the week, we found the same pattern described by Du J. et al. [35]: the average rate of positive tweets was higher on Wednesdays and lower on weekends, while the average rate of negative tweets followed the opposite trend, and the neutral group remained without significant variations throughout the week. In our study, users tweeted more about vaccines during weekdays, especially in the middle of the week, than during weekends. By sentiment, the drop in the absolute number of tweets during weekends was higher in the positive (−44.95%) and neutral group (−39.34%) than in the negative group (−16.14%). This explains why the percentage of negative tweets increases on Sundays and Saturdays, although users write more negative messages in the middle of the week. This should be considered while setting up vaccine promotion interventions.

Among users who wrote more than one vaccine-related tweet containing one of the hashtags included in this study, the majority (91.83%) produced content with a homogeneous opinion towards vaccination in all their discourse. This result is correspondent with Schmidt et al.’s [30] findings on Facebook. Thus, we identified three groups of users with extremely polarised opinions, which is not representative of the vaccine hesitancy continuum described in the general population [1]. We could conclude that social network users and the general population express their opinions differently.

Features such as the number of retweets or favourites/likes a tweet gets are useful to measure engagement. Unlike favourites/likes, retweets have an impact on the spread of the information. In our dataset, positive tweets had higher engagement than negative tweets, and both groups had statistically significantly more retweets and favourites/likes than neutral tweets. The engagement according to sentiment polarity has great variations depending on the different datasets. For example, Blankenship et al. [36] reported that negative tweets had more retweets than positive tweets in a sample of tweets with the #vaccine hashtag, while Massey et al. [37] found that positive tweets about human papillomavirus vaccine had a slightly higher average number of retweets per tweet than negative tweets. Consistent with our findings, neutral tweets had fewer retweets than positive and negative tweets in both datasets.

Sentiment polarity showed significant variations depending on the location. The highest proportion of positive tweets in Switzerland could be explained by the fact that many international organisations have their headquarters in that country and actively use Twitter to promote vaccines. Vaccine hesitancy was more prevalent in the Netherlands, Canada, Japan, and the United States than in the other locations. Like vaccine confidence studies on the general population report [38,39], negative sentiment was not predominant in any country. Nevertheless, the distribution of vaccine hesitancy by country varied significantly from their findings. This highlights our theory that Twitter users are not comparable to the general public. However, our country analysis is probably biased by the selected languages (only English and Spanish) and the location-retrieving method. An exhaustive analysis of the online opinion in a specific country must consider the local language and use more precise geolocation methods, as user’s profile-based locations are self-reported and may be unreal.

There were other limitations in this study. First, the dataset may be incomplete as Tweets were not collected in real-time and, over the years, several messages could have been deleted and some accounts could have been suspended or become private. Second, irony and sarcasm identification is still a challenge for both manual and automatic classification methods, so the results may not be 100% accurate. However, the large number of tweets included in the study reduces the effect of this limitation in the overall results. Third, the texts were labelled for polarity without taking into consideration the context, since the associated images, emoticons, and URL’s were deleted. These items could be added to the analysis to improve the accuracy of the algorithm. Fourth, our analysis was limited to tweets tagged with some preselected hashtags, so the results may not be extrapolated to tweets containing other hashtags and non-tagged tweets. Lastly, Twitter users might not be representative of the general population. Hence, an extrapolation of the results cannot be performed. Instead, social network analysis should not be applied as an independent tool to address vaccine hesitancy, but it must be a component of broader strategies that include both online and offline interventions.

## 5. Conclusions

Social media platforms have become a common source of information and disinformation on vaccines. Public health professionals and organisations should take this into account and include social media monitorisation in their strategies to fight against vaccine hesitancy. The increasing number of vaccine-related tweets over time makes it essential to use automatic or semi-automatic methods for data extraction and text classification, as alternatives to manual coding. Our study highlights the importance of relying on machine learning for opinion-mining in big data and the need to keep improving the algorithms to overcome the present challenges, such as the identification of irony and sarcasm. The application of artificial intelligence has allowed us to perform a fast and low-cost analysis of sentiment polarity towards vaccination in a large number of tweets written over several years. The same study would have used more time and resources if it had been done on the general population, using traditional methods to gather the information (such as surveys) and coding all the data manually.

Unlike most of the previous research, we performed an aspect-level SA instead of a sentence-level SA to improve the precision of the classification. Besides, we did not limit our study to a specific point in time. The inclusion of a long time frame in the study has allowed us to prove that Twitter users usually maintain the same stance towards vaccinations over the years, which has been studied before on Facebook, but not in long-term research on Twitter. In addition, we observed that there is an increasing proportion of polarised tweets over the years. This underlines the need to implement continuous vaccine-hesitancy surveillance on social media. The information extracted by this kind of monitorisation could be useful to build and adapt vaccine promotion strategies.

Our research also emphasises the value of geolocating the messages, as the proportion of posts by sentiment polarity are very different among the countries. By doing so, sentiment analysis in Twitter and other social networks could be useful for public health professionals as an auxiliary tool to early identify locations where vaccine hesitancy may be most prevalent or increasing and may benefit from an intervention.

Future research might analyse the predominant topics in positive and negative vaccine-related tweets in our dataset. We will also investigate if there is any correlation between sentiment polarity by vaccine product and vaccines coverages in some countries.

## Data Availability

Data available on request due to privacy or ethical restrictions.

## Appendix A

Twitter basics:

Microblogging platforms are systems that allow users to exchange small amounts of content. Twitter is a free microblogging platform launched in 2006. Twitter users have a personal space where they can write messages called tweets. Tweets can contain images, links to web pages and texts, limited to 140 characters from 2006 to October 2017, and 240 characters from November 2017 until the present. Each tweet has a unique identification number (tweet ID).

Each Twitter user is identified by a unique username of his or her choice, composed of the symbol @ followed by a maximum of 15 characters. Users can change their usernames anytime they want. They also have a unique identification number (user ID), given automatically by Twitter when they register on the platform. User ID always remains the same regardless of the times the user changes his or her username.

Twitter users can become followers of other accounts to get notifications whenever they post a new tweet. Twitter users can set their accounts to private or public. The content of private accounts can only be seen by followers. The content posted on public accounts can be seen by anyone on the Internet, even people that do not have a Twitter account. Twitter has a search box where individuals with and without an account can look for usernames. They can also search for keywords and hashtags to find tweets regarding a particular subject.

In the following paragraphs, we provide definitions for some relevant Twitter terms:

Hashtags: Labels composed of the symbol # followed by letters or numbers without spaces between them. Users can add these labels to their messages to categorize them by topics. By clicking on a hashtag in a tweet, an individual can see the list of tweets that have been tagged with the same hashtag. Hashtags are not predetermined by Twitter and users can create any hashtags they want.

Favourites or likes: Twitter users can indicate they appreciate a tweet by clicking on the heart icon underneath it. Favourites were replaced with “likes” in November 2015.

Retweet: A retweet is a re-posting of a tweet. A user can retweet his or her tweets or other user’s tweets. Retweets begin with “RT” or have the icon  followed by the name of the user who retweeted the message and the word “Retweeted”.

Reply: A direct response to another person’s tweet. A Twitter user can write a reply by clicking on the speech bubble icons underneath a tweet. A reply begins with the username of the author of the tweet being replied to. If the sender account is private, the reply can only be seen by his or her followers.

Mention: A tweet containing another account’s Twitter username anywhere except at the beginning of the tweet. The usernames included in replies and mentions are links to those people’s Twitter profiles.

Twitter Application Programming Interface (API): An API is a set of functions and procedures that allows two programs to communicate with each other. Twitter API is the intermediary between a third-party application and Twitter platform that allows users to create and retrieve data from the Twitter database.

## Appendix B

Preliminary study search terms: vaccine * OR immune * OR antivax * OR getvax OR vax * OR vacuna * OR inmuniza * OR antivacun *.

## Appendix C

Main study search terms: #antivaxx, #antivaxxer, #getvax, #immunization, #learntherisk, #vaccinate, #vaccinateyourkids #vaccination #vaccinations #vaccine #vaccinefailure #vaccinehesitancy #vaccineinjury #vaccinesafety, #vaccineskill #vaccines #vaccinesdontwork #vaccinessavelives #vaccineswork #vaxxed, #antivacunas #inmunizacion #lasvacunasfuncionan #lasvacunassalvanvidas #vacuna #vacunas #vacunacion #vacunate.

## Appendix D

Sentiment polarity classification criteria:

Positive: tweets supporting vaccination or sending messages against vaccine hesitancy.

Negative: vaccine-hesitant tweets.

Neutral: any tweet that did not match the criteria for positive or negative categories. This includes tweets that express no sentiment (objective tweets) and tweets whose sentiment cannot be understood without a further context.
